# Enhancing Cardiopulmonary Resuscitation Training: An Interprofessional Approach With Undergraduate Medicine and Nursing Students Using Self-Learning Methodology in Simulated Environments (MAES)—A Qualitative Study

**DOI:** 10.1155/jonm/9470402

**Published:** 2024-12-19

**Authors:** Giulio Fenzi, Carolina Alemán-Jiménez, Lucia López-Ferrándiz, Cesar Leal-Costa, José Luis Díaz-Agea

**Affiliations:** ^1^Nursing Department, Catholic University of Murcia (UCAM), Murcia, Spain; ^2^Nursing Department, University of Murcia (UM), Murcia, Spain

**Keywords:** cardiopulmonary resuscitation, clinical simulation, focus group analysis, interprofessional education, self-learning methodology

## Abstract

**Background:** Training the knowledge and skills needed to recognize and respond quickly to cardiac arrest significantly increases patient survival rates. Recent advances in life support training focus on simulations. Interprofessional education, involving collaborative training between nursing and medical students, would enhance mutual understanding of roles and perspectives, resulting in comprehensive and real-world learning experiences. Self-learning methodology in simulated environments actively engages students by combining problem-based learning with clinical simulation.

**Objectives:** To analyze the perspective of a group of nursing and medical students in interprofessional training with self-learning methodology in simulated environments, clinical simulation in cardiopulmonary resuscitation, as well as to describe the main strengths and weaknesses detected and worked on during cardiopulmonary resuscitation training with the self-learning methodology in simulated environments.

**Method:** This multicenter qualitative study, based on focus group analysis, delves into the views of nursing and medical students who underwent interprofessional clinical simulation training in cardiopulmonary resuscitation.

**Results:** Benefits such as increased interest, motivation, and sense of responsibility are highlighted, along with improvements in teamwork, interprofessional education, and acquisition of both technical and nontechnical skills. Participants valued the structured debriefing sessions, which allowed them to learn from their mistakes. Suggestions for improvement revolved around the time constraints and responsibilities inherent in the methodology.

**Conclusions:** Interprofessional cardiopulmonary resuscitation training using the self-learning methodology in simulated environments offers an innovative and effective approach to improve traditional cardiopulmonary resuscitation training paradigms.

## 1. Introduction

Cardiac arrest continues to represent a public health problem with a high morbidity and mortality rate. It is necessary to emphasize the importance of correct care provided at an early stage, known as the “chain of survival.” Specific training for healthcare personnel in charge of attending to these emergency situations is essential to improve patient survival [1].

There are numerous clinical practice guidelines that have been modified over the years, which contain the latest recommendations for cardiac arrest situations. Two of these guidelines come from the American Heart Association (AHA) [2] standing out in the USA, and the European Resuscitation Council (ERC) in Europe [3]. These recommendations on cardiopulmonary resuscitation (CPR) highlight the importance of early identification of this situation, to begin early patient care, thus increasing the chances of recovery of spontaneous circulation. When the healthcare professional knows how to identify a cardiac arrest, and is trained in how to respond, the patient's chances of survival are clearly increased. In this regard, it is vital to know how to recognize this circumstance and this is achieved through prior training [4].

The latest developments in life support training are aimed at recreating a real-life situation so that students can improve their resuscitation skills and increase the survival rate of the population [5]. Simulation-based learning enables healthcare professionals to acquire not only knowledge and technical skills but also clinical reasoning-related abilities and nontechnical skills [6]. Keeping calm and knowing how to communicate with the rest of the team in a stressful simulated situation help both healthcare students and professionals when they witness a real situation. As a consequence, they are sufficiently trained and know how to respond effectively, ensuring maximum safety for the patient [7]. Regular updates on this type of situation are vital to prevent skills or knowledge from being lost over time. Thanks to clinical simulation and technological advances, the simulated experience can offer the maximum realism of the situation. With training in simulated CPR environments, skills can be practiced and improved at the same time as they are being performed.

Clinical simulation is a highly effective teaching method for healthcare practice that is being developed worldwide, with increasingly more realistic simulators and environments. These scenarios allow students to safely practice situations in which they develop skills that will help them in their professional performance [8, 9]. The continuous innovation to improve and create new educational methodologies takes down the boundaries of education. Health education moved its focus from theory in classrooms to practical and simulation methods. It helps to overcome traditional limitations and expand the possibilities for effective teaching and learning. In this scenario, the literature shows us some recent educational methods such as flipped classrooms [10] and in situ simulation [11, 12] developed as a consequence of COVID-19 limitations. In the first instance, students are required to procure adequate materials for their theoretical preparation at home or through preclass assignments. The acquired knowledge must then be applied to a new situation that could be a simulated scenario, as it happens in a flipped classroom simulation. In the second case, the focus shifts to conducting simulation scenarios in a real clinical environment with genuine interprofessional teams and performing a hot debriefing right at the end of the simulation [13]. Simulation with interprofessional teams in as realistic as possible scenarios also sits in the continuously evolving context of clinical simulation methodology. It answers to the need to train students and professionals in realistic teams, and it is called interprofessional education (IPE) with simulation. With this method, students from two or more professions learn about, from and with each other to enable effective collaboration and improve health outcomes [14]. IPE is a methodology that can combine simulation learning and collaborative learning. More specifically, when students from two health professions (nursing and medicine in this case) participate in mixed teams, in a simulated environment and in situations with similar characteristics to those they will experience in their professional future, it allows for mutual learning about the training of colleagues from the other discipline, understanding their functions and their points of view. When nurses and doctor students train together, their learning is more complete and much more in line with reality than traditional learning [15]. Moreover, these training experiences are very well received by the students, who find them very enriching as a team unites with a shared purpose, goal, and a profound mutual respect, all dedicated to delivering safe, high-quality healthcare [16, 17].

Within this panorama, there is the methodology of self-learning in simulated environments (MAES) which was developed in 2013 based on different training models such as problem-based learning, peer education, and clinical simulation in highly realistic environments [18]. The structure of MAES is developed with 6 phases in a minimum of 2 sessions and the completion of homework by the student between sessions 1 and 2. The work consists of the design of the scenario and the search for scientific evidence to support it (Table 1).

MAES consists of involving students with the guidance of a facilitator, an expert in this methodology. The students take on a more active role in their own training. In this methodology are the students themselves who, in small working groups, reflect on the aspects of their training that they need to develop and seek the necessary evidence. Following a structured scheme, they can develop a clinical case and respond to the different situations that may arise during the simulation. With proper guidance from the facilitator, simulation can have a positive impact on students' motivation, as they perceive this learning as more transferable to real practice [19, 20]. Finally, they can discuss with the rest of the group about the case, the knowledge acquired, and the experience lived [18]. The use of MAES as an educational tool provides the student with a feeling of greater motivation [21] learning and making the most of the sessions [20], which results in better academic results and the development of critical thinking [22, 23]. All these aspects ultimately lead to safer healthcare [18, 24].

MAES has been accepted in university and clinical training with nurses [18, 25], but it has not been sufficiently investigated with interprofessional simulation in CPR training. Based on the information described in the literature on the importance of CPR training and the need to implement MAES with CPR and IPE training, the objectives of the present study were to analyze the perspective of a group of nursing and medical students in interprofessional training with MAES clinical simulation in CPR, as well as to describe the main strengths and weaknesses detected and worked on during CPR training with the MAES method.

## 2. Materials and Methods

### 2.1. Study Location and Participants

The study was conducted at the Catholic University of Murcia (UCAM, Spain) and at the Catholic University of Valencia (CUV, Spain) in 2022. The choice of these two centers was due to the similarities between their nursing and medicine degree programs. Students from both the universities engage in simulation-based learning during their studies (a minimum of 20 h of clinical simulation per year). In addition, both nursing degree curricula at UCAM and CUV include MAES simulation (a minimum of 8 h in the third year and fourth year). The students' experience with simulations made them comfortable with procedures, scenarios, and debriefing. Finally, both nursing and medicine students from UCAM and CUV undergo training in basic life support (BLS) CPR following the ERC guidelines [3]. Despite their extensive use of simulation for student training, neither of the two universities has any experience with IPE during their programs.

Participants were chosen based on convenience sampling [26]. As inclusion criteria, the participants had to have previous experiences with simulation and previous training in BLS. They had to be in the last year of nursing (fourth) or medicine (sixth) university. The total number of eligible students between the two centers was 180 (110 nursing students and 70 medicine students). Finally, the participants recruited based on their willingness and availability to be a part of the IPE CPR training with MAES were *N* = 23. Their participation in the training was not compulsory. The reasons for nonparticipation among the remaining students included scheduling conflicts, lack of interest, and prior commitments. They were organized into two groups: 13 in CUV (7 medicine students and 6 nursing students) and 10 in UCAM (5 medicine students and 5 nursing students). The simulation experience with interprofessional CPR MAES training was performed between March (CUV) and July (UCAM) 2022.

### 2.2. Design and Data Analysis

This study employed a descriptive qualitative design [27] based on thematic analysis research [28]. The analysis followed a general inductive approach, allowing themes to emerge naturally from the data [29]. This approach was chosen to investigate students' experiences with a new educational programme. It provided an in-depth understanding of the participants' experiences and perceptions related to the topic of the research and its objectives. It aimed to highlight patterns within the data, rather than testing predefined hypotheses. By using thematic analysis, a rigorous and systematic reading and coding of the transcripts allowed major themes to emerge. The inductive approach facilitated the development of themes based on the raw data, ensuring that the findings were aligned with participants' perspectives.

Data were collected through structured debriefing of the simulated scenarios and focus groups. The use of both methods ensured flexibility to probe deeper into specific areas of interest while maintaining a consistent framework. All debriefings of interprofessional CPR training with MAES were performed at the end of each scenario. It followed the gather–analyze–summarize (GAS) debriefing method [30]. In the analytical phase of the debriefing, the facilitator used the plus/delta method (“plus” are the things that went well and “delta” are the ones that can be improved) [31]. In addition to plus/delta, the participants' mental models were investigated with a deep reflection technique [32]. At the end of each debriefing, the pluses/deltas' information and the mental models were transcribed and organized by consensus in a table visible to all the participants. Graphic recordings have been shown to improve concept fixation, motivation, and critical thinking compared to simply discussing topics without recordings [33]. This method allowed researchers to reduce biases and allowed participants to review and agree with the debriefing's final findings. The focus groups were performed at the end of each simulation session. In both cases (debriefing and focus groups), video and audio recordings were obtained as well as researcher's notes. Photos of the final debriefing tables were also obtained, which summarized the main learning points of the scenario.

The audio/video files, the notes, and the photos of the final tables of plus/delta were recorded, transcribed, and transferred to a single document for analysis. All data were accessible by the researchers. The data analysis process began with familiarization, where researchers read and reread the transcripts. After careful and independent reading by different members of the research team, the final document served as the basis for categorization and thematic analysis [34]. Initial codes were generated to capture significant features of the data. The units of significance that emerged from the independent analysis of the text were identified and coded in different initial themes. After identifying the themes, different subthemes were recognized according to research objectives as a function of their similarity [35].

Throughout the analysis, reflexive practices were employed to acknowledge and address potential biases. The research team engaged in regular discussions to critically examine the emerging themes and ensure a transparent analytical process. The Standard for Reporting Qualitative Research (SRQR) checklist was used as a guide for the analysis of the qualitative data and the conduct of the focus groups [36] (Supporting file 4).

### 2.3. Procedure

The CPR interprofessional clinical simulation training with MAES was developed in two simulation sessions, carefully organized in accordance with MAES methodology [18]. The structure was the same in both universities (two sessions in UCAM and two sessions in CUV).

During the first session, participants were informed about the study's objectives, the simulation development process, and the MAES structure. The sessions were led by an expert MAES facilitator (G.F.) with over 5 years of experience with MAES and more than 7 years as a clinical simulation instructor. The facilitator helped to create small working groups by mixing students from both degree programs. He also ensured the creation of a safe environment to promote group identity and cohesion. The psychological safety of the participants was addressed through group dynamics following Lewin's theory and Tajfel's theory [37] (1: group identity and group factor). The division resulted in three definitive workgroups at each university. The workgroups were organized as follows: two people designed the scenario, two people performed the simulation, and the remaining were observers. In addition, in this session, students selected the main theme of their scenario based on the research of video, news, or newspaper articles about cardiac arrest patients which may directly move their interest (2: choose scenario). Finally, students selected their learning needs and learning objectives based on their intrinsic motivation (3: baseline learning objectives) [38]. Students from CUV wanted to learn the different CPR rhythms and the CPR sequence looking into special cases and focusing on team leadership. On the other hand, students from UCAM wished to better look at a special cardiac arrest case: CPR in drowning patients. They wanted to learn about protocols for drowning patients in cardiac arrest, focusing on managing the situation and possible hypothermia. All the learning objectives chosen by students were designed to complement the theoretical and clinical components of their respective programs by offering practical, hands-on training in CPR and clinical understanding of a cardiac arrest patient.

The participants had a period between sessions to design the scenarios (4: homework) based on the learning objectives set in the prebriefing. They stated them in their scenario tables as “learning goals and discussion points” or “technical & nontechnical skills.” As a result of the learning objective chosen by students, the designed cases were “cardiac arrest secondary to myocardial infarct” (CUV) (see Supporting file 1) and “cardiac arrest secondary to drowning” (UCAM) (see Supporting file 2).

In the second session, students who designed the scenario proceeded by giving a short handover to the group who was performing the simulated experience (5: simulation). At the end of each simulation, the debriefings were conducted following the structured phases of the reflective learning: reaction, analytic, and summary phase (GAS) [30]. In the first phase, participants shared their feelings. In the second phase, they proceed to analyze what went well and what they could improve. Finally, they summarized the important points with the help of the simulation facilitator [39]. The debriefing sessions lasted approximately 45 min each. They were conducted by an experienced MAES facilitator (G.F.), who has over 7 years of experience in conducting debriefings. The facilitator, during his career in clinical simulation, received specialized training in debriefing techniques, including advanced simulation debriefing methods and reflective practice, ensuring a high level of proficiency in guiding participants through the debriefing process. The result of the debriefing was recorded and organized by the facilitator.

At the end of both experiences, participants were asked for their perceptions of the experience through focus group discussion. A total of two focus groups were conducted, one at each university, with all participants attending the focus group at their respective universities. Each focus group session lasted a minimum of 1 h and was led by the researcher G.F. The discussions were guided by 10 questions (Supporting file 3), covering topics such as students' feelings during the IPE MAES sessions, aspects of collaborative work, their opinion on the interprofessional CPR training, the simulation and scenarios they worked on, the IPE experience, and their overall impression of MAES.

The outcomes of MAES sessions were aligned with the ERC guidelines. The scenarios required students from both disciplines to work collaboratively, enhancing interprofessional training, mirroring real-world clinical settings.

### 2.4. Ethical Considerations

No rights were violated, and no data compromised the privacy and safety of any students. All the participants signed an informed consent form before the first MAES session with the possibility of opting out of the study at any time. The simulations were always conducted with respect and promoted the values of unity and teamwork.

To ensure trustworthiness, we employed credibility, transferability, dependability, and confirmability strategies. Researchers performed prolonged engagement, member checking, and triangulation of data sources (focus groups and observations). A detailed description of the research context, participants, and study settings was provided. A comprehensive audit of the research decision and procedures, as well as reflexivity, was maintained. To ensure reflexivity, the research team comprises individuals from different backgrounds who incorporated diverse perspectives, maintained reflective practices, and critically examined assumptions and biases throughout the whole study.

The work was conducted in accordance with the Declaration of Helsinki (1964) and it was approved by the Ethics Committee from the UCAM, with reference number: CE052308. To safeguard the identity of the participants when presenting the verbatim text, the researcher assigned a code to the participants (S1: Student 1, S2: Student 2, etc.).

## 3. Results

In our study, we included a total of 23 participants. The participants' ages ranged from 21 to 26, with a mean age of 24.5 years. The sample consisted of 3 males (13%) and 20 females (87%). The educational backgrounds of the participants were similar: the medical students were all in their sixth year of medical school, and the nursing students were all in their fourth year of nursing school. In Spain, the fourth and sixth years correspond to the final years of nursing and medical degrees, respectively. Additionally, all the fourth-year nursing students had already completed their Critical Care or Emergency Care traineeship placements before participating in the study. Only four students had previously seen a real cardiac arrest patient, and only two of them had performed cardiac compressions in real life. This comprehensive demographic profile aids in understanding the representativeness of our sample and supports the interpretation of the study results.

**Figure 1:**
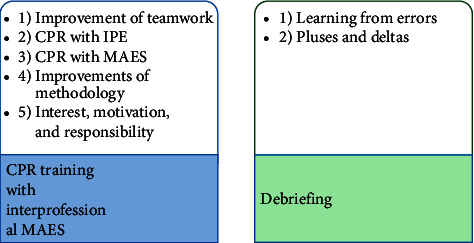
Conceptual map. Themes and subthemes of the study.

The results of the inductive analysis of the study can be organized into 2 main themes and 7 different subthemes (Figure 1).

### 3.1. CPR Training With Interprofessional MAES

The first main theme resulting from our thematic analysis highlighted some of the characteristics that cardiac arrest training with MAES implements from the participant's point of view. The fact of performing interprofessional CPR training helped them to improve and train both technical and nontechnical skills (teamwork). It enhanced the awareness of other health professionals present in those emergencies before they could face a real situation. They also highlighted the effectiveness of the MAES in student engagement. They emphasized the benefits of realistic scenarios, shared responsibilities, and a structured debriefing process, which collectively contribute to improved nontechnical skills, personal growth, and knowledge retention. While the methodology is well-received, some students identified areas for improvement, such as the need for more time in scenario design and managing the responsibility of peer learning. Overall, the approach fosters a deeper connection to real-world medical scenarios and promotes collaborative learning.

#### 3.1.1. Improvement of Teamwork

The MAES methodology significantly enhances teamwork in interprofessional settings, as students noted its positive impact on scenario design, simulation, and debriefing, fostering better collaboration and nontechnical skill development. Working with the MAES methodology in interprofessional teams was seen by most of the students as a support to improve teamwork. This was reflected in the whole process, both in the design of the scenarios and in the simulation debriefing.

*“The dynamics help us to get to know each other better and to work as a team. We can train nontechnical skills that with other methodologies may not get the same attention.”* (S3)

*“The methodology seems to humanise the teaching. The possibility of interprofessional MAES training makes the team stronger, which is reflected in the simulation team when acting on the patient*.” (S5)

*“The presence in the scenario of medicine and nursing allows us to give more reality to the scene and above all each participant takes more responsibility. We don't have to simulate decisions that we wouldn't make in our jobs. It improves teamwork and indirectly allows us to improve our specific skills.”* (S11)

#### 3.1.2. CPR With IPE

The students, by training with an interprofessional experience, got a more similar dynamic to the one they will face at work. The participants associated the possibility offered by the methodology with an enriching novelty. They commented that they saw cardiorespiratory arrest situations as possible critical situations in their future jobs. Training medical and nursing students together was perceived not only as a possibility of theoretical and practical preparation but also as an improvement on a personal and human level. Interprofessional training fosters collaboration between medical and nursing students, enriching their perspectives, enhancing real-world readiness, and improving skills crucial for their future work environments.

*“I liked sharing the experience between medicine and nursing students, because in a few months we will be working together. We had never trained together before in our studies.”* (S12)

*“It changes the student's perspective which is different from what I am used to see and study. I can relate to a more work-like dynamic and share experiences. I think it is very enriching on a personal and human level.”* (S13)

*“Working with this methodology allows us to improve aspects of PCR that go beyond the techniques and that we will face in our work tomorrow*.” (S8)

#### 3.1.3. CPR Training With MAES

Most of the participants highlighted the importance of the CPR training. Their comments were mainly focused on the training experience with different methodologies comparing the possibility to learn both technical and nontechnical skills. It emerged that learning CPR with MAES gave them the possibility to feel in charge of their learning as well as the sensation of being able to retain more knowledge. The MAES methodology places students at the center of their CPR training, enhancing knowledge retention and providing a comprehensive understanding that goes beyond technical skills. They felt MAES was a useful methodology and made them feel more comfortable acting in an emergency compared to CPR lessons and SBL CPR training.

*“Throughout the university we train technical skills on CPR. Sometimes we have the possibility to put these skills into practice with scenarios made by the teacher, but I always have the feeling that we don't manage to relate knowledge. With MAES this did not happen. We felt that we were at the center of our learning, and it allowed us to retain more knowledge about the topic we were dealing with.”* (S19)

*“With MAES I have the feeling of learning CPR, whereas when we do it without MAES it seems that we only focus on the technique. When we go to a patient in cardiac arrest, we cannot only know how to do compressions or ventilations, we also need to have theoretical knowledge and know how to relate it to our patient's situation. Simulating with MAES I have the feeling of receiving a broader knowledge and I feel more prepared.”* (S21)

#### 3.1.4. Improvements of the Methodology

The MAES methodology is valued for its engaging approach to CPR training, though students find the responsibility and additional workload between sessions challenging, especially during busy periods. The participants not only underlined positive aspects of CPR training with MAES IPE, but they also shared possible improvements of the interprofessional MAES methodology. A limit they saw was the short time for the scenario design in between sessions and with a busy calendar. Also, some of them agreed that they felt scared of the responsibility of leaving knowledge to their peers.

*“I like the learning model, but the work at home between the two sessions can be a limit, especially if it is done in a busy period.”* (S15)

*“Learning CPR with MAES is very interesting because it gives you responsibility. Sometimes the responsibility of being the one who helps my classmates to learn such an important subject scare me.”* (S5 and S17)

#### 3.1.5. Interest, Motivation, and Responsibility

The possibility to choose the topic of their simulation scenario from interesting information founded in a newspaper or a video was seen as one of the positive aspects of the methodology [18]. This practice improved interest and increased motivation as students saw the connection between a real fact and their simulated scenarios. In addition, the participants felt more responsible for the deepening of CPR topics.

*“The fact of deciding the scenario from a newspaper, a video or a title, generates more interest and curiosity in the person who chooses. It increases the motivation to face the chosen topic.”* (S2)

*“Being able to choose the topic or how we approach it when designing the scenario, allows us to address CPR issues. With the classic methodology we don't feel we are facing or analysing properly the scientific evidence. We feel more responsible towards the knowledge that would otherwise remain superficial.”* (S6)

### 3.2. Debriefing

The debriefing process in the interprofessional MAES CPR simulation was positively received by participants. They valued the structured approach for its effectiveness in enhancing knowledge retention and error analysis. Debriefings helped students focus on their knowledge underlining and self-realizing what they know and what they must improve. Participants appreciated their teamwork, communication, and role assignment during the scenarios, which contributed to a well-organized workflow. However, they identified areas for improvement (deltas), including a lack of leadership and gaps in knowledge of advanced life support (ALS) protocols. The deltas impacted decision-making and task prioritization during critical moments. This feedback highlights the strengths of the structured debriefing used in MAES methodology as it allowed participants to learn safely from mistakes and live the simulation experience with more security. All these aspects are reflected in students' feelings of being better prepared for real-world scenarios.

#### 3.2.1. Learning From Errors

The debriefing modality adopted in the CPR simulation with interprofessional MAES was widely accepted by the participants. The analysis phase of the debriefing, compared to other more direct or with feedback typologies, was recognized as positive. Students saw analytical debriefing as a possibility to retain more knowledge, with positive analysis of errors. All of this resulted in a better feeling at the end of the simulated experience. Structured debriefing in MAES provides a supportive environment for learning from mistakes, helping students feel more secure and promoting positive, constructive analysis rather than just critical feedback.

*“Structured debriefing allows learning from mistakes. When debriefing is limited to feedback from the teacher, you end the scenario with a bad feeling, you think everything went wrong.”* (S1, S7, and S10)

*“With MAES you live the experience with more security because you know that in the debriefing the analysis of the mistakes is positive. It allows you to learn from them and you don't feel evaluated, although an evaluation of the skills is carried out.”* (S16)

#### 3.2.2. Pluses and Deltas

**Figure 2:**
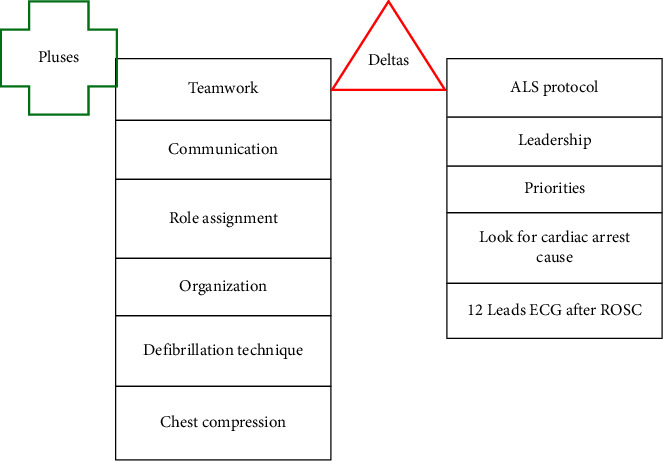
Pluses and deltas resulting from analytic phases of the debriefing.

We have summarized the most frequently discussed aspects in both simulation groups of the 2 participating universities. The results of the two debriefings are shown in Figure 2.

Aspects highlighted in the analysis of the simulation scenarios showed several positive feedbacks. The participants demonstrated excellent teamwork and communication throughout the simulation. Each team member understood their role and contributed effectively to the overall effort, resulting in a cohesive and coordinated response using clear and effective communication. Role assignment in the scenario was also an extremely positive aspect, allowing a smooth workflow. Positive teamwork, communication, and role assignment positively affected the organization of the workflow and the scenario, ensuring that all necessary equipment was prepared and used efficiently. As technical feedback, students underlined the defibrillation technique (correct execution, good placement of the pads, and use of safety protocols as “clear the patient”) and chest compression (high-quality chest compression was performed consistently).

On the other hand, the debriefings highlighted some aspects that could be improved (deltas). In contrast with good teamwork, communication, role assignment, and organization, a lack of leadership was noted. There were moments where ineffective direction and decision-making put patients' safety at risk. This directly affected the prioritization process of the team in critical moments. This caused delays in important tasks such as CPR medication administration following ALS protocols or performing the 12 leads ECG after ROSC. Participants found themselves unsure about the ALS protocols, recognizing their lack of knowledge about the advanced protocols which could have caused delays in their actions and decision-making.

## 4. Discussion

The discussion of our qualitative study focuses on the insights gathered from 23 participants, comprising predominantly female students in their final year of medical and nursing education. The inductive analysis revealed that the MAES methodology significantly enhances interprofessional CPR training by improving both technical (e.g., defibrillation and chest compressions) and nontechnical skills (teamwork, communication, and role assignment), thereby fostering collaboration and increasing student engagement. The IPE MAES methodology ensures learning from errors through deep reflection in its structured debriefing. While students valued the realistic scenarios and structured debriefing, some highlighted areas for improvement, such as the need for more time in scenario design and better leadership during simulations. These findings underscore the importance of refining educational methodologies to better prepare students for real-world medical scenarios.

Currently, the CPR courses of the different institutions (AHA, ERC, UKRC, etc.) respect the models imposed. They are based on the latest recommendations and comply with specific technical and nontechnical skills training [2, 3]. At the same time, as proposed by the latest recommendations of the ERC, there is the possibility for resuscitation courses to be more focused on the individual needs of the trainee. It is in this scenario that the MAES methodology is positioned. It is based on peer education, problem-based learning, and self-directed learning in highly realistic simulated environments [5]. The self-learning methodology has been widely accepted by students and professionals, with a proven improvement of the skills learned in previous studies [21, 25]. Our study is the first to evaluate MAES methodology applied to interprofessional CPR training.

### 4.1. CPR Training With Interprofessional MAES

Recent CPR guidelines recommend training of healthcare personnel in both technical and nontechnical skills [2, 3]. Communication, teamwork, critical situation awareness, and leadership are all crucial factors in achieving high-quality CPR and good clinical practice [5]. As emerges from the literature, training in these factors benefits from interprofessional simulated experiences [4, 40]. The participants in our study had undergone interprofessional simulation training experiences in CPR with MAES. A relevant aspect perceived by the participants in our study is the attention to teamwork. The structure and development of MAES are based on the group factor and the creation of small working groups. This allows participants to develop and train nontechnical skills and group/team relationship skills at every moment of the sessions, without the need for a specific case to work on them [18].

Interprofessional CPR simulation training is essential for enhancing the realism and effectiveness of the learning experience, bridging the gap between classroom theory and real-world practice. By engaging in simulations that involve both medical and nursing students, participants are exposed to a dynamic that reflects the complexities of an actual healthcare environment. This type of training allows students to deal not only with their technical skills (e.g., correct execution of chest compressions and the use of defibrillators) but also to develop crucial nontechnical skills, including teamwork, communication, and decision-making under pressure (improving interprofessional team simulation learning. one more step towards the humanization of health care in [33]). Furthermore, teaching these human and personal factors through realistic, interprofessional simulations not only increases the technical abilities of future healthcare providers but also boosts their confidence and willingness to engage in resuscitation efforts when faced with real emergencies [12].

One of the key outcomes of the methodology used in this study is the enhanced ability of participants to connect their knowledge directly to the patient or the simulated situation. MAES places students at the center of their own learning. This approach enabled them to develop a deeper understanding of their simulated patients and scenarios, allowing them to more effectively relate the information they gathered to the specific circumstances they were simulating [18], all this without leaving aside the objectives of improving technical skills (compressions, ventilations, etc.). With MAES, we also observed an increase in the motivation of the participants. The possibility of choosing the approach to the scenario and the associated learning objectives allowed for greater intrinsic motivation in accordance with previous studies [18]. In the same way, the methodology increased the learners' responsibility towards the search for more up-to-date scientific evidence, with the aim of leaving a higher level of knowledge to peers. Participants associated MAES with the feeling of receiving broader knowledge compared to other methodologies.

### 4.2. Debriefing

In simulation learning, regardless of the methodology involved, a fundamental factor is the debriefing [39]. Using debriefing allows participants to analyze the events of the simulated scenario. In the MAES methodology, an analytical debriefing is involved, which allows both positive aspects and things to be improved to be reflected upon. The analysis structure used allows participants to lose the fear of making mistakes, rather allows them to analyze and learn from them. Moreover, it is carried out with the participation of all the students, which reaches its maximum scope in the expository phase. In this phase, the group that designed the case transmits the knowledge about it, always with the presence of the facilitator as a guide [41]. In accordance with the above, it can be affirmed that with MAES, the students actively participate in the debriefing and they respect the error but lose the fear of declaring it. This makes it possible to become familiar with debriefing and to accustom the health professional to use it for the critical analysis of clinical situations, in accordance with the latest recommendations [1, 2, 13].

The methodology involved in conducting the courses or classes is as important as the content of the courses or classes [40, 42, 43]. In our study, we can observe analogies comparing the results of the “plus delta table” with the students' perceptions emerging from the focus group. The characteristic factors of the MAES methodology, positively perceived by the students (teamwork, communication etc.), were found to be reflected in the pluses of the simulated experiences. The fundamental technical skills in CPR (chest compressions and defibrillation) did not take second place and were also present in the simulation “pluses.” At the same time, technical and nontechnical skills appeared among the aspects to be improved (ALS protocol, looking for cardiac arrest causes, and leadership). These aspects are independent of the MAES methodology. They are associated with more clinical experience and participation in advanced courses, which could be lacking in undergraduate students. In accordance with these findings, the need for continuous training in CPR is once again confirmed as the fundamental aspect for all health professionals [5, 40, 42].

### 4.3. Limitations

The internal validity of the study is assured as all recommended steps in conducting a qualitative study have been followed. Given that this is a qualitative study focused on the opinions of the participants, the results cannot be generalized to other contexts beyond the research itself, as the external validity of this type of study is limited. However, since it is a study performed in two different universities and it has not shown differences in opinion among participants, we can assert that this circumstance would enhance the study's validity.

To consolidate the preliminary results of this study, it would be necessary to increase the number of participants and experiences related to CPR with interprofessional MAES methodology.

## 5. Conclusions

The present study reaffirms the importance of the CPR training. It analyzes a new experience with MAES methodology made by undergraduate nursing and medicine students in CPR training. It demonstrates an overall acceptance of the experience with several positive aspects linked to the MAES methodology applied to IPE training in CPR. Students appreciated the possibility to be the center of their own learning, feeling that they could train both technical and nontechnical skills. They felt more motivation and responsibility towards their peers and their learnings. They also did not feel evaluated with the methodology applied. This fact allowed them to feel less pressure.

The study also shows the positive aspects of the simulation experiences and the things that could be improved. All the positive aspects aligned with MAES, its features, and the latest CPR training guideline standards. Likewise, the areas for enhancement in the clinical simulation experiences were anticipated, given their connection to a more advanced level of competencies. Learning and training CPR with MAES interprofessional could be a new and different approach to propose as an alternative training experience to the usual ones we could find in CPR training. All these aspects are in accordance with the international CPR guidelines, which suggest considering different ways to train, putting learners in the center of the process.

More research is needed to understand better and wider the potential of MAES applied in CPR training and courses.

## Figures and Tables

**Table 1:** MAES sessions and their phases.

Session	Phase	Objective
Session 1	(1) Group identity and group factor	Work groups are established, with a joint identity that is superior to the individual based on values (group essence)
	(2) Choose a scenario	The facilitator presents the students with a group of possible interesting cases. After a discussion session, each team voluntarily chooses the topic to be worked on
	(3) Baseline learning objective	Brainstorm session where all the teams' participants participate and discuss about what they want to learn

Work at home	(4) Design scenario	Each team designs a simulation scenario based on the previously chosen learning objectives

Session 2	(5) Simulation	Simulation experience
	(6) Debriefing	A structured, GAS (gather–analyze–summarize) debriefing. At the end of it, the students provide the information for the rest of the group

## Data Availability

The data used to support the findings of the study are available from the corresponding author upon request.

## Supporting Information

Supporting file 1: MAES case of MI with cardiac arrest. Supporting file 2: MAES case of drowning with cardiac arrest. Supporting file 3: Focus group. Supporting file 4: SRQR.
