# Supplementary material for: Enhancing Cardiopulmonary Resuscitation Training: An Interprofessional Approach With Undergraduate Medicine and Nursing Students Using Self-Learning Methodology in Simulated Environments (MAES)—A Qualitative Study
Source: J Nurs Manag. 2024 Dec 19;2024:9470402. doi: 10.1155/jonm/9470402 (PMC11925266; doi:10.1155/jonm/9470402)
Supplement: Supporting Information — Additional supporting information can be found online in the Supporting Information section. [file 9470402.f1.docx]

**Supplementary files**

**Supplementary file 1**: MAES© case of MI with cardiac arrest.

| **MAES SIMULATION** | | | | | |
| --- | --- | --- | --- | --- | --- |
| **MAES CLINICAL SIMULATION TITLE: ADULT CARDIAC ARREST** | | | | | |
| **TEAM PLANNING THE SIMULATION: EQUIPO** | | | | | |
| **INFORMATION PROVIDED TO THE SIMULATOR GROUP PRIOR TO THE SIMULATION** | | | | | |
| **DEBRIEFING** | | **CLINICAL HISTORY & SITUATION** | | | |
| **LEARNING GOALS & DISCUSSION POINTS** | | **SITUATION** | | | |
| 1. Cardiac arrest rhythm | | Two doctors and two nurses enter in an emergency room where the patient is brought in by the ambulance for chest pain. | | | |
| 2. Special Cases | |  |  |  |  |
| 3. Leadership | |  |  |  |  |
|  | |  |  |  |  |
|  | | **BACKGROUND** | | | |
| **TECHNICAL & NON-TECHNICAL SKILLS** | |  |  |  |  |
|  |  | The ambulance crew stated the family was very nervous and couldn't tell them anything and the patient could not speak due to the pain. | | | |
| 1. CPR sequence | |  |  |  |  |
|  | |  |  |  |  |
|  | |  |  |  |  |
|  | | **ASSESSMENT** | | | |
|  |  | Patient looks unstable, only responds to pain. He is pale/cyanotic. Now is not monitored. | | | |
|  |  | **RECOMMENDATION** | | | |
|  |  | Assessment of the patient and monitorization of vital signs. | | | |
| **CLINICAL HAPPENINGS**  (Evolution of the clinical situation during the simulation) | | | | | |
| While being monitored and peripheral lines are taken, the patient is deteriorating. If an ECG is performed, ST elevation is found in II-III and aVf. If no ECG is performed in the first 2 minutes, the patient goes into arrest (asystole), and if pain medication is administered, it will not be effective. 2) If ECG is performed, after 3 minutes the patient goes into cardiorespiratory arrest with a VF rhythm and CPR is started. 3) After reassessing the rhythm, the patient has ventricular tachycardia. 4) If the cause of the arrest is identified the patient ROSC. 5) If the cause is not identified, the patient goes into arrest rhythm: Asystole. | | | | | |
| **INTERVENTIONS**  (Activities and interventions that the simulator team is expected to perform) | | | | | |
|  | | | | | |
| **INTERVENTION 1: NIC (6140) Management of cardiac arrest** | | | | | |
|  | | | YES | | NO |
| Activity 1: Assess the patient's arrival status and cardiac rhythm. | | |  | |  |
| Activity 2: Monitor the patient and take the necessary lines. | | |  | |  |
| Activity 3: Ensure that the patient's airway is patent and isolate appropriately. | | |  | |  |
| Activity 4: Perform cardio-respiratory resuscitation with a focus on chest compressions in adults and compressions with breathing efforts in children, as appropriate. | | |  | |  |
| Activity 5: Perform cardioversion or defibrillation, as soon as possible. | | |  | |  |
|  | | | | | |
| **INTERVENTION 2: (6320) Resuscitation** | | | | | |
|  | | | YES | | NO |
| Activity 1: Assess the patient's lack of response to determine the appropriate action. | | |  | |  |
| Activity 2: Call for help if breathing is absent or absent and there is no response. | | |  | |  |
| Activity 3: Switch on the AED and carry out the specified actions. | | |  | |  |
| Activity 4: Initiate 30 chest compressions at a specified rate and depth, allowing full chest recovery between compressions, minimising interruptions in compressions and avoiding excessive ventilation | | |  | |  |
| Activity 5: Monitoring the patient's response to resuscitation efforts | | |  | |  |
|  | | | | | |
| **RESOURCES** | | | | | |
|  | Mannequin and Actors  We will need a manikin that can be monitored, heart rates can be changed, CPR can be performed, and lines can be taken. | | |  |  |
|  |  | | |  |  |
|  | Materials  The simulation takes place in an emergency room, so hospital equipment is needed, such as needles for IVs, ventilator, equipment to intubate and monitor the patient, medication, defibrillator, gloves, gauze, drips. | | |  |  |

**Supplementary file 2**: MAES© case of drowning with cardiac arrest.

| **MAES SIMULATION** | | | | | |
| --- | --- | --- | --- | --- | --- |
| **MAES CLINICAL SIMULATION TITLE: RCP AND DROWNING** | | | | | |
| **TEAM PLANNING THE SIMULATION: LAS DEL SUR** | | | | | |
| **INFORMATION PROVIDED TO THE SIMULATOR GROUP PRIOR TO THE SIMULATION** | | | | | |
| **DEBRIEFING** | | **CLINICAL HISTORY & SITUATION** | | | |
| **LEARNING GOALS & DISCUSSION POINTS** | | **SITUATION** | | | |
| 1. Types of drowning. | | 23-year-old male celebrating with friends at a beach bar. The beach has no lifeguard post. The patient is intoxicated when he enters the water, once in the water he cannot swim. His friends realised that he was drowning and went in after him and managed to pull him out. Once out they call EMERGENCY SERVICES. They begin cardiopulmonary resuscitation with the help of EMERGENCY SERVICES by telephone until the emergency team arrives on the beach. | | | |
| 2. Time of cardiac arrest reanimation in drowning and hypothermia. | |  |  |  |  |
| 3. Drowning cardiac arrest protocol. | |  |  |  |  |
|  | |  |  |  |  |
|  | | **BACKGROUND** | | | |
| **TECHNICAL & NON-TECHNICAL SKILLS** | |  |  |  |  |
|  |  | Unknown | | | |
| 1. Managing the situation (patient and environment). | |  |  |  |  |
|  | |  |  |  |  |
|  | |  |  |  |  |
|  | | **ASSESSMENT** | | | |
|  |  | Unresponsive, no signs of life and no carotid pulse palpable. | | | |
|  |  | **RECOMMENDATION** | | | |
|  |  | Assessment ABCDE. Perform advanced cardiopulmonary resuscitation. | | | |
| **CLINICAL HAPPENINGS**  (Evolution of the clinical situation during the simulation) | | | | | |
| Rescue the victim with special care assessing the possible mechanism of injury.● Collect information on previous mobilisation of the victim.● Collect information on circumstances related to drowning: drugs consumed, history of epilepsy, previous loss of consciousness, primary arrhythmia, etc. ● Initial assessment: ABCDE, with special attention to airway maintenance and neurological status.● Start CPR: in the case of a hypothermic patient, prolong CPR until a temperature > 32ºC - 35ºC is achieved. In the drowning patient, rescue ventilations should be performed as a priority, followed by chest compressions if these are not effective.● Monitor if possible: BP, HR, RF, ECG, SatO2 and temperature.● Administer oxygen therapy with a mask to maintain a SatO2 between 94-96%.● Put a venous line.● Monitor body temperature to control the onset of hypothermia: remove wet clothing.● If the patient is drowning, perform external warming.● If the patient is drowning, perform CPR and CPR. If progressing unfavourably, perform endotracheal intubation. | | | | | |
| **INTERVENTIONS**  (Activities and interventions that the simulator team is expected to perform) | | | | | |
|  | | | | | |
| **INTERVENTION 1: Medication administration: intravenous (i.v.) 2314** | | | | | |
|  | | | YES | | NO |
| Activity 1: Follow the five principles of medication | | |  | |  |
| Activity 2: Prepare the appropriate concentration of i.v. medication from an ampoule or vial. | | |  | |  |
| Activity 3: Assess the patient to determine the response to medication. | | |  | |  |
| Activity 4: Verify i.v. catheter placement and patency in vein | | |  | |  |
| Activity 5: Maintaining the sterility of the open i.v. system | | |  | |  |
|  | | | | | |
| **INTERVENTION 2: Emergency Care 6200** | | | | | |
|  | | | YES | | NO |
| Activity 1: Act quickly and methodically, providing care in the most urgent conditions | | |  | |  |
| Activity 2: Perform cardiopulmonary resuscitation, if appropriate | | |  | |  |
| Activity 3: Move the patient to a safe place, if possible | | |  | |  |
| Activity 4: Monitoring vital signs | | |  | |  |
| Activity 5: Maintain body alignment if spinal injuries are suspected. | | |  | |  |
|  | | | | | |
| **RESOURCES** | | | | | |
|  | Mannequin and Actors  We will need a manikin that can be monitored, heart rates can be changed, CPR can be performed, and lines can be taken. The maniking should be placed next to water or next to a blanket which simulate the water. | | |  |  |
|  |  | | |  |  |
|  | Materials  Resus cardiac back, resus airway bag, difficult airway bag, monitor, cannulation materials, medication for Cardiac arrest management | | |  |  |

**Supplementary file 3:** Focus Group.

| **Focus Group Questions (Guide)** |
| --- |
| Have you got any clinical simulation experience? |
| Have you got any Interprofessional education experience? |
| What do you think about teamwork? |
| What do you think about the relation that exist between nursing and medical students on a formation point of view? |
| What do you think about the possibility to work with a doctor/nurse teammate?  What do you think about CPR training?  What do you think about teamwork between doctor and nurses in a CPR situation? |
| Did you know MAES©? |
| Do you think MAES© can help interprofessional training? Can you motivate your answer?  Do you think MAES© and interprofessional training can help CPR training? Why yes/Why not? |

**Supplementary file 4:** SRQR.

|  |  | **Reporting Item** | **Page Number** |
| --- | --- | --- | --- |
|  | [#1](https://www.goodreports.org/srqr/info/#1) | Concise description of the nature and topic of the study identifying the study as qualitative or indicating the approach (e.g. ethnography, grounded theory) or data collection methods (e.g. interview, focus group) is recommended | 6,7 |
|  | [#2](https://www.goodreports.org/srqr/info/#2) | Summary of the key elements of the study using the abstract format of the intended publication; typically includes background, purpose, methods, results, and conclusions | 1 |
| Problem formulation | [#3](https://www.goodreports.org/srqr/info/#3) | Description and significance of the problem / phenomenon studied: review of relevant theory and empirical work; problem statement | 2,3,4,5 |
| Purpose or research question | [#4](https://www.goodreports.org/srqr/info/#4) | Purpose of the study and specific objectives or questions | 5 |
| Qualitative approach and research paradigm | [#5](https://www.goodreports.org/srqr/info/#5) | Qualitative approach and guiding theory if appropriate; identifying the research paradigm is also recommended; rationale. The rationale should briefly discuss the justification for choosing that theory, approach, method or technique rather than other options available; the assumptions and limitations implicit in those choices and how those choices influence study conclusions and transferability. As appropriate the rationale for several items might be discussed together. | 5,6 |
| Researcher characteristics and reflexivity | [#6](https://www.goodreports.org/srqr/info/#6) | Researchers' characteristics that may influence the research, including personal attributes, qualifications / experience, relationship with participants, assumptions and / or presuppositions; potential or actual interaction between researchers' characteristics and the research questions, approach, methods, results and / or transferability | 5,6,8 |
| Context | [#7](https://www.goodreports.org/srqr/info/#7) | Setting / site and salient contextual factors; rationale | 5 |
| Sampling strategy | [#8](https://www.goodreports.org/srqr/info/#8) | How and why research participants, documents, or events were selected; criteria for deciding when no further sampling was necessary (e.g. sampling saturation); rationale | 5 |
| Ethical issues pertaining to human subjects | [#9](https://www.goodreports.org/srqr/info/#9) | Documentation of approval by an appropriate ethics review board and participant consent, or explanation for lack thereof; other confidentiality and data security issues | 8 |
| Data collection methods | [#10](https://www.goodreports.org/srqr/info/#10) | Types of data collected; details of data collection procedures including (as appropriate) start and stop dates of data collection and analysis, iterative process, triangulation of sources / methods, and modification of procedures in response to evolving study findings; rationale | 6 |
| Data collection instruments and technologies | [#11](https://www.goodreports.org/srqr/info/#11) | Description of instruments (e.g. interview guides, questionnaires) and devices (e.g. audio recorders) used for data collection; if / how the instruments(s) changed over the course of the study | 7,8 |
| Units of study | [#12](https://www.goodreports.org/srqr/info/#12) | Number and relevant characteristics of participants, documents, or events included in the study; level of participation (could be reported in results) | 5 |
| Data processing | [#13](https://www.goodreports.org/srqr/info/#13) | Methods for processing data prior to and during analysis, including transcription, data entry, data management and security, verification of data integrity, data coding, and anonymisation / deidentification of excerpts | 6 |
| Data analysis | [#14](https://www.goodreports.org/srqr/info/#14) | Process by which inferences, themes, etc. were identified and developed, including the researchers involved in data analysis; usually references a specific paradigm or approach; rationale | 6 |
| Techniques to enhance trustworthiness | [#15](https://www.goodreports.org/srqr/info/#15) | Techniques to enhance trustworthiness and credibility of data analysis (e.g. member checking, audit trail, triangulation); rationale | 7,8 |
| Syntheses and interpretation | [#16](https://www.goodreports.org/srqr/info/#16) | Main findings (e.g. interpretations, inferences, and themes); might include development of a theory or model, or integration with prior research or theory | 9-13 |
| Links to empirical data | [#17](https://www.goodreports.org/srqr/info/#17) | Evidence (e.g. quotes, field notes, text excerpts, photographs) to substantiate analytic findings | 9-13 |
| Intergration with prior work, implications, transferability and contribution(s) to the field | [#18](https://www.goodreports.org/srqr/info/#18) | Short summary of main findings; explanation of how findings and conclusions connect to, support, elaborate on, or challenge conclusions of earlier scholarship; discussion of scope of application / generalizability; identification of unique contributions(s) to scholarship in a discipline or field | 1,14-16 |
| Limitations | [#19](https://www.goodreports.org/srqr/info/#19) | Trustworthiness and limitations of findings | 16 |
| Conflicts of interest | [#20](https://www.goodreports.org/srqr/info/#20) | Potential sources of influence of perceived influence on study conduct and conclusions; how these were managed | 17 |
| Funding | [#21](https://www.goodreports.org/srqr/info/#21) | Sources of funding and other support; role of funders in data collection, interpretation and reporting – no funding | 17 |
